# Effect of Body Mass Index on pregnancy outcomes in nulliparous women delivering singleton babies

**DOI:** 10.1186/1471-2458-7-168

**Published:** 2007-07-24

**Authors:** Sohinee Bhattacharya, Doris M Campbell, William A Liston, Siladitya Bhattacharya

**Affiliations:** 1 Dugald Baird Centre for Research on Women's Health, Aberdeen Maternity Hospital, Aberdeen, AB25 2ZD, UK; 2 Department of Obstetrics & Gynaecology, Aberdeen Maternity Hospital, Foresterhill, Aberdeen, AB25 2ZD, UK; 3 The Simpson Centre for Reproductive Health, Royal Infirmary of Edinburgh – Little France, Edinburgh, EH16 4SA, UK

## Abstract

**Background:**

The increasing prevalence of obesity in young women is a major public health concern. These trends have a major impact on pregnancy outcomes in these women, which have been documented by several researchers. In a population based cohort study, using routinely collected data, this paper examines the effect of increasing Body Mass Index (BMI) on pregnancy outcomes in nulliparous women delivering singleton babies.

**Methods:**

This was a retrospective cohort study, based on all nulliparous women delivering singleton babies in Aberdeen between 1976 and 2005. Women were categorized into five groups – underweight (BMI < 20 Kg/m^2^), normal (BMI 20 – 24.9 Kg/m^2^) overweight (BMI 25 – 29.9 Kg/m^2^), obese (BMI 30 – 34.9 Kg/m^2^) and morbidly obese (BMI > 35 Kg/m^2^). Obstetric and perinatal outcomes were compared by univariate and multivariate analyses.

**Results:**

In comparison with women of BMI 20 – 24.9, morbidly obese women faced the highest risk of pre-eclampsia {OR 7.2 (95% CI 4.7, 11.2)} and underweight women the lowest {OR 0.6 (95% CI 0.5, 0.7)}. Induced labour was highest in the morbidly obese {OR 1.8 (95% CI 1.3, 2.5)} and lowest in underweight women {OR 0.8 (95% CI 0.8, 0.9)}. Emergency Caesarean section rates were highest in the morbidly obese {OR 2.8 (95% CI 2.0, 3.9)}, and comparable in women with normal and low BMI. Obese women were more likely to have postpartum haemorrhage {OR 1.5 (95% CI 1.3, 1.7)} and preterm delivery (< 33 weeks) {OR 2.0 (95% CI 1.3, 2.9)}. Birthweights less than 2,500 g were more common in underweight women {OR 1.7 (95% OR 1.2, 2.0)}. The highest risk of birth weights > 4,000 g was in the morbidly obese {OR 2.1 (95% CI 1.3, 3.2)} and the lowest in underweight women {OR 0.5 (95% CI 0.4, 0.6)}.

**Conclusion:**

Increasing BMI is associated with increased incidence of pre-eclampsia, gestational hypertension, macrosomia, induction of labour and caesarean delivery; while underweight women had better pregnancy outcomes than women with normal BMI.

## Background

The rising rate of obesity is a major public health concern in the West, where 28% of pregnant women are overweight and 11% are obese [1]. In the United States, the incidence of obesity in pregnancy varies from 18.5% to 38.3% according to the definition used [2-5]. In the UK, 56% of all women are over the recommended BMI, with 33% of them classified as overweight (BMI > 25) and 23% obese (BMI > 30). Although the exact incidence of obesity in pregnant women in the UK is not known, the Confidential Enquiry into Maternal and Child Health 2004 [6] reported that 35% of all maternal deaths occurring in the triennium 2000–2002 were in obese women with Body Mass Index > 30 Kg/m^2^. Pregnancy complications in overweight women were studied as early as 1945 [7]. Since then, a number of studies have reported a clear association between maternal overweight and adverse obstetric and perinatal outcomes. Data from North America have been supported by results from Danish [8,9] and Swedish studies [10,11]. In the UK, Sebire [12] studied the effects of maternal obesity on pregnancy outcomes in a London cohort of 287,213 women. Since then, similar reports have been published from Wales [13] and Scotland [14].

The effect of maternal underweight on obstetric performance is less clear. While some researchers [4,15] have found increased incidences of preterm delivery, low birth weight and increased perinatal loss in these women, others [16] have reported a protective effect of maternal underweight on certain pregnancy complications and interventions.

Definitions of overweight, obesity and underweight differ in the different reports. In earlier research the relationships between maternal height and weight with pregnancy complications were extensively explored, but in recent times, Body Mass Index (BMI) is widely accepted as a better measure of over or underweight. More recently, the waist-hip ratio has been used to study the effects of obesity on pregnancy, but data relating to this parameter are seldom available. Despite the plethora of publications on obesity and obstetric outcomes, population based studies in the UK reporting on the effect of extremes of Body Mass Index (high as well as low BMI) on pregnancy outcomes are relatively few.

The Aberdeen Maternity and Neonatal Databank (AMND) has recorded information on all pregnancy related events occurring in Aberdeen city and district since 1950, and currently contains over 200,000 such records. The height and weight of women as well as the gestational weeks at the first antenatal visit have been systematically logged, thus offering a unique opportunity to study the effect of Body Mass Index (BMI) on pregnancy outcomes.

The aim of this study was to examine the association between Body Mass Index (BMI) and obstetric and perinatal outcomes in primigravid women delivering singleton babies.

## Methods

This was a retrospective cohort study using data from the Aberdeen Maternity and Neonatal Databank (AMND). Ethical approval was granted by the North of Scotland Research Ethics Committee  for all observational studies using routinely collected anonymised data from the AMND, provided permission was granted by the Steering Committee (Caldicott guardians) of the AMND. After obtaining permission from the Caldicott guardians, data were extracted from the Aberdeen Maternity and Neonatal Databank on all primigravid women delivering singleton babies after 24 weeks of gestation in Aberdeen city and district between 1976 and 2005. Women who booked after 16 weeks were excluded. In the dataset, 7.2% of the women did not have either their height or weight or both recorded and were excluded from the analysis. Missing data on outcome variables varied from 1.2 – 2.8%. These women were also excluded. Missing outcome data were distributed evenly among the various BMI categories. All variables recorded in the AMND are coded using the ICD-9 codes and several consistency checks are in place to ensure data quality. Opportunistic checks carried out in the past using surveys and linkages with larger databases have shown the records to be 85 to 90% complete [17]. Sociodemographic variables extracted included age at delivery, height and weight measured and recorded at antenatal booking visit, husband or partner's social class (recorded according to the Register General's classification), smoking, marital status and pre-existing Type I diabetes mellitus. Body Mass Index was calculated using the formula weight/height^2^. The women were then categorised into five groups according to their BMI as follows after Abrams [18].

Underweight: less than or equal to a BMI of 19.9 Kg/m^2^

Normal: BMI of 20 – 24.9 Kg/m^2^

Overweight: BMI of 25 – 29.9 Kg/m^2^

Obese: BMI of 30 – 34.9 Kg/m^2^

Morbidly obese: BMI greater than 35 Kg/m^2^

The group with BMI in the normal range (20 – 24.9 Kg/m^2^) was used as the reference or comparison group for the analysis.

Obstetric outcomes included the following: pre-eclampsia, gestational hypertension and antepartum haemorrhage due to placenta praevia or abruptio placentae, type of labour (ie spontaneous or induced), the type of delivery (ie spontaneous vaginal, instrumental or Caesarean section), preterm delivery (37 weeks and 34 weeks). Perinatal outcomes included stillbirth rate and birthweight. The total number of Caesarean sections and the number of elective sections were recorded in the AMND – the number of emergency sections was calculated by subtracting the second from the first number. Gestational age was recorded according to the last menstrual period throughout the database and was confirmed by ultrasound ever since it became available from 1986 onwards. In case of discrepancy, the ultrasound date was taken as the actual gestational age.

Statistical analysis was conducted using Statistical Package for Social Scientists (SPSS version 14). The anonymised dataset was extracted by AMND staff and given to named researchers who conducted the analysis. Univariate analysis was done using ANOVA or Mann-Whitney test for continuous variables and chi-square test for categorical variables. A P-value of less than 0.05 was regarded as statistically significant. Potential confounders were adjusted for using logistic regression. The risks of obstetric complications were presented as crude and adjusted Odds Ratios with 95% confidence intervals.

## Results

**Figure 1:**
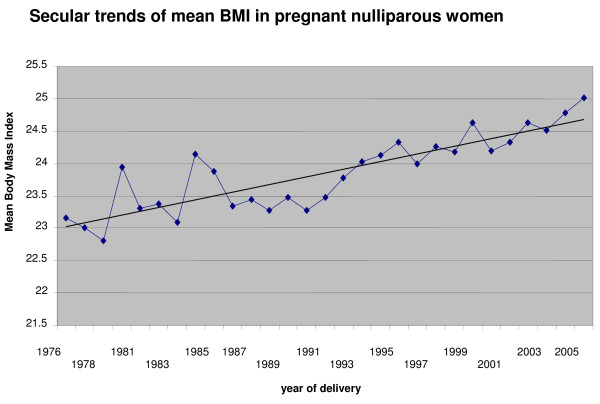
Trends in mean BMI, Aberdeen city & district 1976–2005.

A total of 24,241 women were included in the study. Of these, 2,842 (11.7%) were underweight, 14,076 (58.1%) had normal BMI, 5,308 (21.9%) were overweight, 1,858 (7.7%) were obese and 157(0.6%) were morbidly obese. Figure 1 shows the increasing trends in the prevalence of obesity in the study population over time.

A comparison of the sociodemographic characteristics of the women in the five BMI categories is presented in Table 1. Women in the underweight category were significantly younger (mean age 24.8, SD 5.2) while those in the morbidly obese group were significantly older (mean age 28.3, SD 5.3) than those with normal BMI (mean age 26.4, SD 5.2). The mean ages of overweight and obese women were comparable with women with normal BMI. As we included all women who had booked at the Antenatal Clinic up to 16 weeks of gestation, we compared the time of booking and found no differences among the different BMI categories. Fewer women were married or cohabiting in the underweight {2201 (77.4%)} and morbidly obese {125 (79.6%)} groups in comparison with women with normal BMI {11848 (84.2%)}. Fewer women in the abnormal BMI categories were from higher socioeconomic groups (as indicated by their partner's social class I/II) in comparison with women with normal BMI {3771 (26.8%)}. This difference in social class was more marked in the groups of obese {358 (19.3%)} and morbidly obese {24 (15.3%)} women. The prevalence of Type I diabetes was higher in the morbidly obese group {3 (1.9%)} as compared to the normal group {27 (0.2%)}. Smoking was significantly more common in the underweight group.

**Table 1:** Sociodemographic characteristics of women in the different BMI groups

Characteristics	Underweight BMI < 20 n = 2842	Normal BMI = 20–24.9 n = 14076	Overweight BMI = 25–29.9 n = 5308	Obese BMI = 30–34.9 n = 1858	Morbidly Obese BMI > 35 n = 157
Age at delivery (years)	24.8 (5.2)*	26.4(5.2)	26.8(5.2)	26.8(5.1)	28.3(5.3)*
Height (cm)	163.0(6.5)	162.5(6.3)	162.2(6.4)	162.2(6.4)	162.4(6.0)
Weight (Kg)	50.1(4.6)	59.2(5.7)	70.9(6.7)	87.3(9.5)	114.1(11.2)
BMI (Kg/m^2^)	18.8 (0.9)	22.4(1.3)	26.9(1.4)	33.2(2.5)	43.3(3.7)
Booking week	10.7(2.6)	10.8(2.6)	10.6(2.7)	10.2(2.7)	9.7(2.8)
Married or cohabiting	2201(77.4%)*	11848(84.2%)	4449(83.8%)	1522(81.9%)	125(79.6%)*
Husband/partner's social class I/II	662(23.3%)*	3771(26.8%)	1227(23.1%)*	358(19.3%)*	24(15.3%)*
Smoking	1208(42.5%)*	4832(34.3%)	1691(31.9%)*	568(30.6%)*	32(20.4%)*
Type I Diabetes	0(0%)	27(0.2%)	40(0.8%)*	5 (0.3%)	3(1.9%)*

Values expressed as mean (SD) median (IQR) or number (per cent)

* P value less than 0.05

Table 2 shows the incidence of complications of pregnancy, labour and delivery in women in the five BMI categories, while Table 3 presents the risk of each complication or intervention in the abnormal BMI categories in comparison with the normal. Both pre-eclampsia and gestational hypertension increased linearly with increasing BMI, resulting in an adjusted Odds Ratio of 7.2 (95% CI 4.7, 11.2) for pre-eclampsia and 3.1 (95% CI 2.0, 4.3) for gestational hypertension in the morbidly obese category when compared to those of normal BMI. Being underweight seemed to have a protective effect on the development of pregnancy induced hypertension – adjusted OR 0.6 (95% CI 0.5, 0.7) for pre-eclampsia and 0.7 (95% CI 0.6, 0.8) for gestational hypertension. A similar relationship was observed with regard to placental abruption, but as the numbers were small, the Odds Ratios had overlapping confidence intervals. The incidence of placenta praevia was not significantly different in the different BMI categories, with the highest proportion occurring in underweight women {10 (0.4%) vs 25 (0.2%) in the normal BMI group}.

**Table 2:** Pregnancy, labour and delivery characteristics of women in each BMI group

Characteristics	Underweight BMI < 20 n = 2842	Normal BMI = 20–24.9 n = 14076	Overweight BMI = 25–29.9 n = 5308	Obese BMI = 30–34.9 n = 1858	Morbidly Obese BMI > 35 n = 157
Pre-eclampsia*	82(3.3%)	572(5.0%)	313(8.1%)	181(14.7%)	29(28.2%)
Gestational hypertension*	374(13.6%)	2662(19.7%)	1422(28.5%)	624(37.2%)	54(42.2%)
Abruptio placentae	12(0.4%)	83(0.6%)	31(0.6%)	13(0.7%)	3(1.9%)*
Placenta praevia	10(0.4%)	25(0.2%)	11(0.2%)	3(0.2%)	0(0%)
Induced labour*	683(24.0%)	3832(27.2%)	1771(33.4%)	796(42.8%)	77(49.0%)
Instrumental delivery	773(27.2%)	4040(28.7%)	1499(28.2%)	465(25.0%)	38(24.2%)
Total C-Section*	320(11.3%)	2305(16.4%)	1279(24.1%)	573(30.8%)	67(42.7%)
Elective C-section	73(2.6%)	487(3.5%)	221(4.2%)	88(4.7%)*	16(10.2%)*
Emergency CS*	247(8.7%)	1818(12.9%)	1058(19.9%)	488(26.3%)	51(32.5%)
					
Mean blood loss (mls)*	254.5 (6.9)	290.4 (5.7)	333.6(8.5)	400.5(7.2)	456.4(25.7)
Postpartum haemorrhage*	192(6.8%)	1356(9.5%)	708(13.3%)	369(19.9%)	37(23.6%)
Preterm delivery (< 37 weeks)	345(12.1%)*	1537(10.9%)	573(10.8%)	243(13.1%)*	32(20.4%)*
Preterm delivery (< 33 weeks)	58(2.0%)	331(2.4%)	122(2.3%)	77(4.1%)*	10(6.4%)*
Spontaneous preterm(< 37 wks)	244(8.5%)*	964(6.8%)	325(6.1%)	124(6.6%)	12(7.6%)*
Post term delivery (> 41 weeks)	108(3.8%)*	773(5.5%)	350 (6.6%)*	136(7.3%)*	7(4.5%)
Stillbirth	22(0.8%)	131(0.9%)	57(1.1%)	35(1.9%)*	4(2.5%)*
Birth weight < 2500 g	269(9.5%)*	980(7.0%)	327(6.2%)	142(7.6%)	19(12.1%)*
Birth weight > 4000 g*	100(3.5%)	1072(7.6%)	564(10.6%)	255(13.7%)	25(15.9%)

Values expressed as number (per cent) or mean (Std. dev.)

*P value less than 0.05

**Table 3:** Crude and adjusted risks of obstetric complications in the different BMI groups compared to normal (OR 1)

Characteristics	OR (95% CI)	Underweight BMI < 20 n = 2842	Overweight BM I= 25–29.9 n = 5308	Obese BMI = 30–34.9 n = 1858	Morbidly Obese BMI > 35 n = 157
Pre-eclampsia*	Crude	0.7(0.5–0.8)	1.7(1.4–1.9)	3.3(2.7–3.9)	7.4(4.8–11.5)
	Adjusted	0.6(0.5–0.7)	1.6(1.2–1.8)	3.1(2.8–3.5)	7.2(4.7–11.2)
Gestational hypertension*	Crude	0.6(0.5–0.7)	1.6(1.5–1.8)	2.4(2.2–2.7)	3.0(2.1–4.2)
	Adjusted	07(0.6–0.8)	1.5(1.4–1.7)	2.2(2.1–2.6)	3.1(2.0–4.3)
Induced labour*	Crude	0.8(0.7–0.9)	1.3(1.2–1.4)	2.0(1.8–2.2)	2.6(1.9–3.5)
	Adjusted	0.8(0.8–0.9)	1.3(1.2–1.4)	1.8(1.6–2.0)	1.8(1.3–2.5)
Elective C-section	Crude	0.7(0.6–0.9)*	1.2(1.0–1.4)	1.4(1.1–1.8)*	3.2(1.9–5.3)*
	Adjusted	0.8(0.6–1.0)	1.1(0.9–1.3)	1.4(1.0–1.8)	3.1(1.7–6.1)*
Emergency CS	Crude	0.9(0.9–1.0)	1.7(1.5–1.8)*	2.4(2.1–2.7)*	3.2(2.3–4.5)*
	Adjusted	0.9(0.8–1.1)	1.5(1.3–1.6)*	2.0(1.8–2.3)*	2.8(2.0–3.9)*
Postpartum hge	Crude	0.7(0.6–0.8)*	1.4(1.3–1.6)*	2.3(2.1–2.6)*	2.9(2.0–4.2)*
	Adjusted	0.8(0.7–1.0)	1.1(1.0–1.2)	1.5(1.3–1.7)*	1.3(0.8–1.9)
Preterm delivery (< 37 weeks)	Crude	1.1(0.9–1.3)	1.0(0.9–1.1)	1.2(1.1–1.4)*	2.1(1.4–3.1)*
	Adjusted	1.0(0.9–1.3)	1.0(0.9–1.1)	1.2(1.0–1.4)	1.6(1.0–2.7)
Preterm delivery (< 33 weeks)	Crude	0.9(0.7–1.2)	1.0(0.8–1.2)	1.8(1.4–2.3)*	2.8(1.4–5.4)*
	Adjusted	0.9(0.7–1.1)	1.0(0.8–1.1)	2.0(1.3–2.9)*	2.0(0.8–4.9)
Spont. Preterm (< 37 weeks)	Crude	1.4(1.1–1.9)	0.8(0.6–1.0)	0.9(0.5–1.1)	1.3(1.1–2.8)
	Adjusted	1.4(1.1–1.9)	0.8(0.6–1.1)	1.0(0.5–1.2)	1.2(1.1–2.8)
Post term (> 41 weeks)	Crude	0.7(0.6–0.8)*	1.2(1.1–1.3)*	1.4(1.1–1.6)*	0.8 (0.4–1.7)
	Adjusted	0.9(0.7–1.1)	0.9(0.8–1.1)	0.9(0.7–1.1)	0.8(0.4–1.8)
Stillbirth	Crude	0.8(0.5–1.3)	1.2(0.9–1.6)	2.0(1.4–3.0)	2.8(1.0–7.6)
	Adjusted	1.0(0.6–1.6)	1.3(0.9–1.9)	1.8 (1.1–2.9)*	1.1(0.3–4.1)
Birth weight < 2500 g	Crude	1.4(1.2–1.6)*	0.9(0.7–1.0)	1.1(0.9–1.3)	1.8(1.1–3.0)*
	Adjusted	1.7(1.2–2.0)*	0.9(0.7–1.1)	1.1(0.9–1.3)	0.7(0.4–1.5)
Birth weight > 4000 g*	Crude	0.4(0.4–0.6)	1.4(1.3–1.6)	1.9(1.7–2.2)	2.3(1.5–3.5)
	Adjusted	0.5(0.4–0.6)	1.4(1.3–1.6)	1.9(1.6–2.2)	2.1(1.3–3.2)

*P value less than 0.05

All variables adjusted for relevant sociodemographic characteristics and year of delivery; induced labour, preterm delivery and caesarean section also adjusted for pre-eclampsia and gestational hypertension; postpartum haemorrhage for induction of labour and caesarean section; birthweight for gestational age and sex

The frequency of induced labour increased with rising BMI; the risk being lowest in underweight women {OR 0.8 (95% CI 0.8, 0.9)} and highest in the morbidly obese {OR of 1.8 (95% CI 1.3, 2.5)}. Both elective and emergency caesarean sections were more common in the morbidly obese group, but only emergency caesarean section rates were significantly different in the other BMI categories. In contrast to women with normal BMI, women who were morbidly obese had a 3 times (95% CI 1.7, 6.1) higher risk of having an elective caesarean section, and 2.8 times (95% CI 2.0, 3.9) higher risk of an emergency caesarean section. The adjusted Odds Ratios for emergency caesarean section increased with increasing BMI, again with a protective effect seen in underweight women {OR 0.7 (95% CI 0.6,0.8)}.

The risk of postpartum haemorrhage remained statistically significant only in obese women, although mean blood loss following delivery showed a linear increase with increasing BMI.

After adjusting for confounders, the odds of having a preterm delivery, ie a delivery before 37 completed weeks were similar in the different groups. However, obese women faced an increased risk of delivery before 33 completed weeks of gestation {OR 2.0 (95% CI 1.3, 2.9)}. Post term delivery, ie delivery after 41 completed weeks of gestation was similar in the different BMI categories after adjusting for confounders. Among the women who delivered before 37 weeks risk of spontaneous preterm birth was increased in the underweight {adjusted OR 1.4 (95% CI 1.1, 1.9)} and the morbidly obese groups {adjusted OR 1.2 (95% CI 1.1, 2.8)}.

Stillbirth rates were significantly higher in the obese {35 (1.9%)} and morbidly obese {4 (2.5%)} groups as opposed to 131 (0.9%) in the normal BMI group. But this difference did not remain statistically significant in the morbidly obese group after adjusting for confounders.

Although low birth weight (birth weight less than 2,500 g) was more common at the two extreme ends of the BMI categories, this remained significant after adjusting for confounders, only in underweight women who had an Odds Ratio 1.7 (95% CI 1.2, 2.0) compared to normal. Macrosomia (birthweight > 400 g) was more common in the obese and morbidly obese groups with Odds Ratios of 1.9 (95% CI 1.6, 2.2) and 2.1 (95% CI 1.3, 3.2) respectively, compared to the normal BMI group.

## Discussion

This study adds to the increasing body of evidence which suggests that obesity, measured by BMI, predisposes women to complicated pregnancies and increased obstetric interventions. We found a linear relationship between increasing body mass index and the risk of developing pre-eclampsia, gestational hypertension, induction of labour and emergency caesarean section. Conversely, low BMI had a protective effect on some obstetric complications.

Previous research has found a strong association between increasing BMI and pregnancy induced hypertension. A meta-analysis of the risk of pre-eclampsia associated with maternal BMI [19] showed that the risk of pre-eclampsia doubled with each 5 to 7 Kg/m^2 ^increase in prepregnancy BMI. We found a 3 times higher risk of pre-eclampsia in obese (BMI 30 to 39.9 Kg/m^2^) and a 7 times higher risk in morbidly obese (BMI > 40 Kg/m^2^) primigravid women. We also found a significantly lower risk of pre-eclampsia in underweight women {OR 0.6 (95% CI 0.5 – 0.7)}, a finding corroborated by Sebire *et al *[16].

Our results agree with earlier reports which have shown an association between increasing BMI and interventions like induced labour [10,13,20] and caesarean delivery [8,10,14,23]. Some previous work has also demonstrated a strong link between postpartum blood loss and BMI. Although we found a linear increase in mean postpartum blood loss with increasing BMI, the risk of postpartum haemorrhage, defined as blood loss of more than 500 ml for vaginal delivery and 1000 ml for caesarean delivery, was significantly higher only in the obese category. Other studies have reported conflicting results. While Sebire *et al *[12] observed a 70% increase in postpartum haemorrhage, Bianco *et al *[22] found no such difference in the incidence. As measurement of blood loss is subjective, and the definition of postpartum haemorrhage variable, it is difficult to make comparisons across studies. Intuitively, it appears that women with higher body mass index should bleed more, but this is at least in part due to the increased incidence of induced labour and operative deliveries in these women.

In contrast to the majority of studies in the literature [21,16] our adjusted data failed to show any differences in the risk of preterm delivery (delivery before 37 completed weeks) in the different BMI categories. Cnattingius [11] found no association between preterm delivery before 37 weeks and prepregnancy weight, although the risk of very preterm delivery before 33 weeks was increased in overweight nulliparous women. This was corroborated by our results, which showed that the risk of preterm delivery before 33 weeks was higher in the obese group, but not in the morbidly obese. On the other hand, Sebire *et al *[12] found that delivery before 32 weeks was significantly less likely in the obese.

With regard to intrauterine growth retardation measured by the adjusted birth weight, we found a strong association with maternal BMI. While the risk of low birth weight (birth weight less than 2,500 g) was higher in underweight women, macrosomia was much more common in the obese and morbidly obese groups. Several studies investigating the relationship of maternal obesity with fetal growth have shown that obese women have an 18 – 26% increased chance of delivering large for date infants, even after controlling for maternal diabetes [12,22-24].

Yu *et al *[25] suggest that the rapid fetal growth induced by maternal hyperinsulinaemia coupled with placental insufficiency may result in the antepartum demise of the fetus in obese pregnant women. Indeed this hypothesis has been corroborated by several epidemiological studies [9,10]. This study found an increased risk of stillbirth in obese, but not morbidly obese women. In reality, there were too few women in the morbidly obese group to comment on this group's association with a rare outcome like stillbirth.

Apart from the slightly increased risk of having a baby with low birthweight, the mothers with BMI < 20 appeared to be at a lower risk of developing any other pregnancy or labour complications compared even to women with BMI in the normal range – a finding corroborated by Sebire *et al *[16]. Several complications like postpartum haemorrhage, preterm delivery and macrosomia were found to increase linearly with rising BMI, but no longer remained statistically significant in the morbidly obese women after adjusting for confounders. This can be partially explained by the smaller sample size of morbidly obese women and partially by the overwhelming effect of pre-eclampsia, gestational hypertension and interventions during labour and delivery in these women.

The growing interest in obesity in pregnancy has prompted at least two good quality reviews [25,26] and several primary studies. Most studies have used a retrospective cohort design using data from routinely collected hospital databases [12,21] or trial data (Weiss – FASTER trial) [24]. In neither case do the data reflect population trends. As the AMND records and stores information on all pregnancy events in a geographically defined area, our data set is truly population based. We have restricted our data between 1976 and 2005, as the principal aim of this study was to examine pregnancy outcomes while minimising the effects of changes in clinical practice over time. Nevertheless, even this limited dataset, shows a rising incidence of obese women booking for antenatal care. Despite restricting this study to nulliparous women delivering singleton babies we achieved a sample size of 24,241 women, which to our knowledge makes it one of the largest studies of this kind. In contast with most retrospective studies, all pregnancy events were concurrently recorded by AMND staff thereby limiting recall bias. The height and weight recorded at the booking antenatal visit were coded using stringent criteria and standard definitions and are subject to validity and consistency checks, making the data completely reliable. More information about coding and quality of the dataset is available at the AMND website [27].

This study, like any other observational study of its kind suffers from several limitations. Firstly, the ideal time to record the baseline height and weight of a pregnant woman is before she has started gaining weight due to gestation. As this is seldom available on a routine database, most researchers have relied on the woman's recall of her pre-pregnancy height and weight, the reliability and standardisation of which is very doubtful [28]. In our study we have relied on height and weight recorded in early pregnancy, before any real impact of gestational weight gain. Still, values recorded in early pregnancy remain an approximation of the pre-pregnancy weight, and therefore subject to bias. Also, exclusion of all women who booked after 16 weeks of gestation could have resulted in selection bias, overweight or underweight women being systematically excluded from the dataset. However, we found an even distribution of the week of antenatal booking visit amongst the different BMI categories, thereby minimising selection bias. Our study used data collected over 30 years, during which time there have been several changes in obstetric protocols, especially with regard to induction of labour and caesarean deliveries and this may have influenced some of the outcomes studied. To account for this, we included year of delivery in the logistic regression model when deriving the adjusted Odds Ratios.

Recent reviews [29] on obesity and pregnancy have highlighted several issues relevant to research and management policy. Firstly, the lack of standard definitions of overweight and obesity makes comparison of findings across studies difficult. While most reports define obesity as an increased body mass index of greater than or equal to 30 Kg/m^2 ^(IOM), others have defined it as increased waist circumference, increased waist – hip ratio or body weight of more than 90 Kg. This makes comparison of studies difficult and may have implications in the management of normal pregnancy, as in the United States, recommended gestational weight gain is dependent on women's prepregnancy BMI categories [30]. Moreover, in most clinics, pre-pregnancy BMI is not recorded routinely, thereby making extrapolation of booking weight or women's recall of prepregnancy weight unreliable. Krishnamoorthy *et al *[29] suggest that all pregnancies in obese women be acknowledged as high risk and managed according to strict guidelines. Management should include prepregnancy counselling to reduce weight; shared antenatal care and appropriate management of complications. The evidence for obesity as an important complication in pregnancy is mounting; it is time to inform practice based on this evidence.

## Conclusion

Maternal BMI shows strong associations with pregnancy complications and outcomes. Obesity is associated with increased incidence of pre-eclampsia, gestational hypertension, macrosomia, stillbirth, induction of labour and caesarean delivery; while underweight women appear to have better pregnancy outcomes than even women with BMI within the normal range.

## Competing interests

The author(s) declare that they have no competing interests.

## Authors' contributions

SB^¥ ^was responsible for designing the study, analysing the data and writing the first and final drafts. DC and WAL conceived of the original research idea and were responsible for facilitating data extraction from the Aberdeen Maternity and Neonatal Databank. SB had input into design of the study, analysis plan, interpretation of the results and editing the first draft. All four authors contributed to the writing of the final draft.

## Pre-publication history

The pre-publication history for this paper can be accessed here:


